# Greater body mass index is related to greater self-identified cold tolerance and greater insensible body mass loss

**DOI:** 10.1186/s40101-016-0105-7

**Published:** 2016-08-22

**Authors:** Dahee Jung, Dami Kim, Joonhee Park, Joo Young Lee

**Affiliations:** 1 College of Human Ecology, Seoul National University, Seoul, South Korea; 2 Research Institute of Human Ecology, Seoul National University, Seoul, South Korea

**Keywords:** Insensible perspiration, Insensible body mass loss, Thermal tolerance, Body mass index, Body weight, Psychological polymorphism

## Abstract

**Background:**

Insensible body mass loss (IBL) from the human body continuously occurs, which is an important component in body heat exchange. The purpose of this study was to examine the relevance of IBL to anthropometric characteristics and self-identified thermal tolerance.

**Methods:**

A total of 289 healthy young Korean males were chosen and sorted into the following three groups: heat tolerable only (HTO, *N* = 79), cold tolerable only (CTO, *N* = 104), neither heat nor cold tolerable (NHC, *N* = 106). They weighed before and after a 30-min rest under lightly clothed condition at an air temperature of 23 ± 1 °C with a relative humidity 55 ± 5 %RH.

**Results:**

(1) The IBL of 289 males had a mean of 90 ± 75 g h^−1^ (48 ± 40 g h^−1^ m^−2^); (2) No significant difference in IBL among the three groups were found; (3) Significant differences in body weight and body mass index (BMI) among three groups were found (*P* < 0.05), but insignificance was found for height (*P* = 0.726) or body surface area (*P* = 0.059); (4) CTO was approximately 4.1 kg heavier in body weight (*P* < 0.05) and higher in BMI (*P* < 0.01) than in HTO; (5) Only for the group CTO, IBL (g h^−1^) showed a positive relationship to BMI (*P* < 0.05, *R*^2^ = 0.056), but there was no relationship between IBL and body surface area.

**Conclusions:**

For healthy young males within normal anthropometric ranges in Korea, IBL was positively related to BMI, and individuals with greater BMI showed greater self-identified cold tolerance, but no direct relationship was found between IBL and self-identified cold tolerance. This suggests that body physique (e.g., BMI) could be an explanatory factor between insensible body heat loss and subjective cognition on cold tolerance.

## Background

The vaporization of water from the skin is an important component in thermal homeostasis. Insensible perspiration has been the classical subject of scientific investigations for over 80 years. In general, insensible body mass loss (IBL) is composed of the vaporization of water through the skin and respiratory passages and the amount of inspired oxygen and expired carbon dioxide through the respiratory tract, which can be expressed algebraically as follows: IBL = H_2_O + CO_2_ − O_2_. Hourly, total insensible body mass loss from a normal healthy adult resting in a comfortable environment has been reported as approximately 23 g h^−1^ m^−2^ [1] (0.45~1.9 kg per day for an adult with 1.8 m^2^ in body surface area), of which 60 % emanates from the skin and 40 % from the respiratory tract. Because 580 kcal of evaporative heat is lost when 1 kg of water evaporates through the skin and the average percentage of body heat loss through vaporization of water varied from 23 to 27 % [2], it is tentative but reasonable to assume that individuals with greater insensible perspiration than others might get experienced greater body heat loss in daily life, which might affect establishing self-identified thermal tolerance over time.

The insensible perspiration is influenced by external and internal factors such as air temperature [3–5], air humidity [1, 5, 6], posture [7], activity [2, 8–10], circadian rhythm [11], skin temperature [9, 12], body region [13], metabolism [1, 5], body fluid [8], obesity [14], burned skin [15], age [16], and diseases [14]. Higher air temperature, lower humidity, and higher skin temperature cause greater IBL [3, 4, 6, 9, 12]. Also, the palm and the head had greater regional IBL than the chest or arms [13]. Furthermore, insensible perspiration is known as a significant index for estimating total heat production of normal adults under a basal state [17]. Although such anthropological and physiological significance of IBL in human heat exchange, very little information are available concerning relationships to anthropometric characteristics, such as body weight, height, or body surface area. If body surface area is greater, IBL could be greater, which induces greater body heat loss by evaporation from the skin. In this regard, it is reasonable to hypothesize that greater insensible body mass loss can cause a greater sense of being cold in daily life under the assumption of that body heat loss by convection and radiation maintained.

An individual who perceive cold more intensively (or who consider themselves cold sensitive) in a thermal environment compared to others can be considered as an individual who is subjectively less tolerable to cold. A term, “self-identified cold tolerance” was defined as subjective cold tolerance which is identified by an individual [18]. This may not be in agreement with physiologically evaluated cold tolerance, but it is important that self-identified thermal tolerance drives thermoregulatory behavior. From perspectives on physiological polymorphism in thermoregulation [19], classifying individuals with their self-identified thermal tolerance might be considered as psychological polymorphism in behavioral thermoregulation. Investigating the relevance of the absolute quantity of IBL and self-identified thermal tolerance is an explorative attempt to expand fundamental knowledge on human thermal regulation.

In this regard, the purpose of this study was to investigate the relationships between anthropometric characteristics and insensible body mass loss for clothed adult males at a comfortable environment. Secondly, we investigated the relevance between self-identified thermal tolerance and IBL. We hypothesized that (1) body mass index would have positive relationships with insensible body mass loss or self-identified cold tolerance and (2) there would be a positive relationship between insensible body mass loss and self-identified cold tolerance.

## Methods

### Subjects and survey

**Fig. 1:**
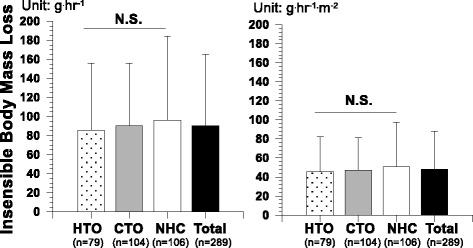
Insensible body mass loss per hour (*left*) and per hour and body surface area (*right*) of three groups: heat tolerable only (*HTO*), cold tolerable only (*CTO*), and neither heat nor cold tolerable (*NHC*)

A total of 289 healthy young Korean males (mean ± SD: 23.8 ± 4.7 years in age, 175.4 ± 5.5 cm in height, 70.4 ± 10.8 kg in body weight, 1.89 ± 0.17 m^2^ in body surface area, 23 ± 3 in body mass index (BMI)) participated in the measurement (Table 1). Among 436 male students who initially volunteered, only the following three groups classified by self-identified thermal tolerance were selected for the measurement: heat tolerable only (*HTO*) (I am tolerable to heat only; *N* = 79), cold tolerable only (*CTO*) (I am tolerable to cold only; *N* = 104), neither heat nor cold tolerable (*NHC*) (I am intolerable to both heat and cold; *N* = 106). Another group, *HCT* (I am tolerable to both heat and cold; *N* = 8), were excluded because of the small number of responses. Furthermore, 139 individuals who expressed as being in the middle between “tolerable” and “intolerable” to heat or cold were excluded because of greater efficiency for the investigation between IBL and the self-identified thermal tolerance. No statistical differences between three groups were found in age, height, and body surface area (BSA) (Table 1). However, CTO had a significantly greater body weight and BMI when compared to HTO (Fig. 1, *P* < 0.05). We interviewed all subjects about thermal behavior and thermal preference in their daily life using a questionnaire which was introduced by Park and Lee [18]. According to the questionnaire, NCH preferred more auxiliary heating devices, thermal gloves, hats, or thermal underwear in winter when compared to the CTO, whereas the CTO sweated a lot in summer, especially on the face, preferred winter to summer, and require air-conditioning system in summer [20]. The categorized groups were confirmed by the thermal behavior from the questionnaire results. This study was approved by the SNU Institutional Review Board (IRB #E1503/002-007).

**Table 1:** Anthropometric characteristics of subjects

Group	HTO (*N* = 79)^a^	CTO (*N* = 104)^b^	NHC (*N* = 106)^c^	Total (*N* = 289)	*P*
Age (year)	23.9 ± 2.9	23.6 ± 6.6	23.7 ± 3.3	23.8 ± 4.7	0.924
Height (cm)	175 ± 5	175 ± 6	176 ± 6	175 ± 6	0.726
Body weight (kg)	68.5 ± 8.6a	72.6 ± 9.4b	70.5 ± 10.5ab	70.7 ± 9.9	0.013
BMI^d^	22.3 ± 2.3a	23.6 ± 2.7b	22.8 ± 2.8a	23.0 ± 2.7	0.002
BSA (m^2^)^e^	1.87 ± 0.13	1.91 ± 0.13	1.89 ± 0.15	1.89 ± 0.14	0.059

“a,” “b,” and “ab” represent statistically classified groups by ANOVA

^a^HTO: I am tolerable to heat only

^b^CTO: I am tolerable to cold only

^c^NHC: I am intolerable to both heat and cold

^d^BMI (body mass index) = body weight (kg)/height (cm)^2^

^e^BSA (body surface area) = height (cm)^0.725^ × body weight (kg)^0.425^ × 72.46

### Measurements

Measurements were conducted during the spring (May) in Korea. We provided participants with guidelines about hydration, nutritional status, physical condition, sleeping requirement, and drinking restriction a couple of days before the measurement day. We also guided participants to come wearing their long sleeve shirts, long pants, underpants, socks, and running shoes (about 0.66 clo in total thermal insulation estimated by ISO 9920 [21]). The materials of upper garments were also restricted in 100 % cotton or cotton with polyester. For lower garments, participants wore their cotton trousers, denim jeans, or training pants. There were no clothes in leather, fur, or plastic materials. Participants refrained from diet and exercise for at least 2 h prior to the measurement. Only drinking water for 2 h was allowed to avoid a non-hydrated state. Measurements were conducted from AM 1000 to PM 1800 except lunch time. All participants were under a controlled environment with an air temperature of 23 ± 1 °C and air humidity of 50 ~ 60 %RH. No one feel any sense of being cool or warm inside the room. They were weighed before and after a 30-min rest using an electronic balance (Sartorius Company, Germany, Sensitivity 1 g), and we doubled the mass change for 30 min to convert to a 60-min change. During the 30-min rest on a chair, they were restricted from having any foods or drinks, urinating, and sleeping, which could cause errors in body weight measurement.

### Statistical analysis

All data were expressed as mean and standard deviations (mean ± SD). Correlation coefficients between continuous variables were calculated using SPSS V22.0. ANOVA was conducted to test differences between the three groups in terms of age, height, body mass, body surface area, body mass index, and IBL. To test a moderator effect of the variable (insensible body mass loss) on the self-identified cold tolerance, we conducted a regression analysis. A statistical significance was set at *P* < 0.05.

## Results

**Fig. 2:**
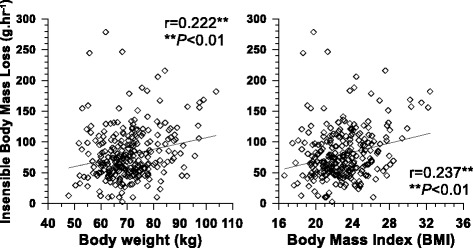
Relationships between insensible body mass loss and body weight (*left*) or body mass index (*right*) (*N* = 289)

**Fig. 3:**
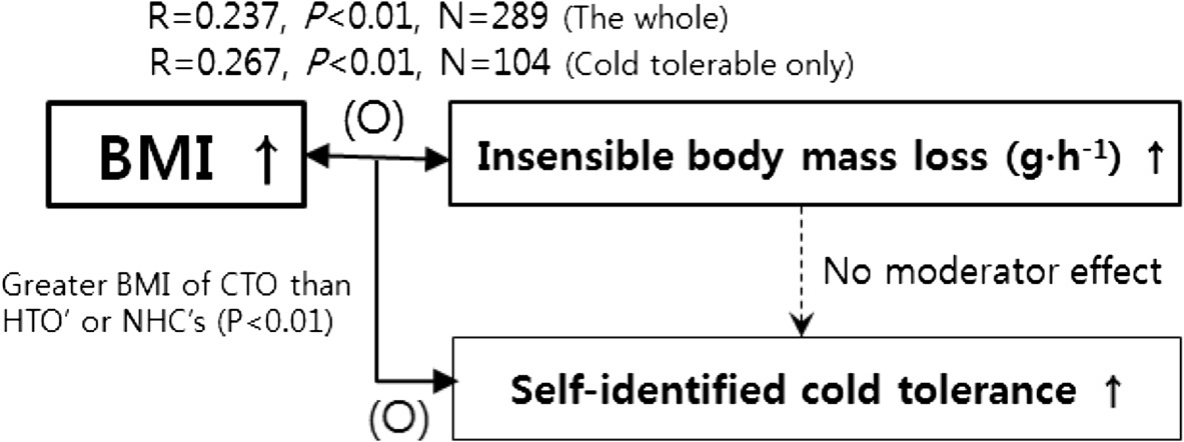
Relationships between body mass index (*BMI*), insensible body mass loss, and self-identified cold tolerance

IBL of the 289 young males had a mean of 90 ± 75 g h^−1^ (48 ± 40 g h^−1^ m^−2^). Although the group NHC showed greater IBL than other two groups and the IBL of HTO was the least with 85 ± 70 g h^−1^ (51 ± 47 g h^−1^ m^−2^), no significant difference was found among the three groups (Fig. 1). While IBL had no relationships with age and height, IBL had significant relationships with body weight or BMI showing that the greater body weight, the greater IBL (*r* = 0.222, *P* < 0.01) and greater BMI had greater IBL (*r* = 0.237, *P* < 0.01) (Fig. 2). When we converted IBL (g h^−1^) to the amount of IBL divided by BSA (g h^−1^ m^−2^), no relationship to body weight or BMI was found. In particular, when reanalyzing the data for each group, IBL (g h^−1^) showed a significant relationship to BMI for the CTO only (*r* = 0.267, *P* < 0.01), whereas no relationship was found for HTO and NHC. When we merged Figs. 1 and 2, a tendency was found that the greater BMI had the greater IBL from individuals who were more tolerable to cold (Fig. 3). For the moderator analyses between self-identified thermal tolerance and BMI, the effect of insensible body mass loss was not significant.

## Discussion

The first hypothesis of this study was accepted. We found positive relationships between BMI and IBL (Fig. 2) and greater BMI for the group who were cold tolerable only (Table 1). The second hypothesis was rejected because there was no difference in IBL among the three groups (Fig. 1). However, an interesting finding was that BMI might play the role of a bridge between self-identified cold tolerance and IBL even though no direct relationship was found between self-identified cold tolerance and IBL. BMI had positive relationships to both self-identified cold tolerance and IBL (Fig. 3). This can be interpreted as showing that physical factors, such as body volume or body mass, are significant factors in both insensible perspiration and subjective cold tolerance. Traditionally, it is well known that 24-h basal heat production could be predicted from the hourly basal insensible perspiration based on a linear equation (e.g., cal h^−1^ = 1.325 insensible body mass loss per hour + 19.3; insensible perspiration = 0.91 × insensible body mass loss) [8]. The present results recall the intervention of physical factors into daily heat exchange in a comfortable environment through water vaporization.

The second interesting finding was that the absolute amount of IBL obtained from this study was greater than conventional values that have been reported in previous studies. For example, the average value of IBL in the present study was 90 ± 75 g h^−1^ (48 ± 40 g h^−1^ m^−2^), whereas Kuno [1] reported 23 g h^−1^ m^−2^ of insensible perspiration. Also, Lamke and his colleagues [4] reported that the average total cutaneous insensible perspiration of resting subjects (1.75 m^2^ in BSA) was estimated to be 22 g h^−1^. According to Newburgh and his colleagues [22], healthy young males’ insensible perspiration showed 63~67 g h^−1^. Relatively greater or smaller insensible body mass loss could be related to differences in regular diet, posture, and the amount of loss from the respiratory gas exchange (evaporation from the respiratory tract and difference between *V*O_2_ and *V*CO_2_). Regarding diet, greater respiratory quotients (RQ) (i.e., consumed O_2_ < produced CO_2_) induced greater insensible perspiration and RQ is greater for a carbohydrate-centered diet compared to a fat-centered diet [23]. We did not assess the diet of the participants on the day, but it is assumed that Korean males have a more carbohydrate-centered diet compared to Western male subjects. Secondly, there could be the effect of posture on insensible perspiration. Benedict and Wardlaw [7] found that the insensible water loss increased at an average of 20 % in the sitting position when compared with the lying position. Most previous studies were conducted in the lying position, while our subjects were in the sitting position. Thirdly, we reported insensible body mass loss whereas some of the previous reports calculated “insensible perspiration.” As insensible perspiration forms about 91 % of insensible body mass loss, such a difference should be considered. Through the mass analysis of expired CO_2_ and inspired O_2_, respiratory perspiration could be calculated but one limitation of this study was that respiratory exchange was not measured. Fourthly, there could be the effect of clothing on insensible body mass loss. It is reported that the average gain from clothing was approximately 2 g [14]. We did not provide participants with the experimental clothing but previously guided all participants to wear their long sleeved-cotton shirts and long cotton trousers, so that the covering area (%BSA) and clothing layers made similar between participants. No identical clothing for all participants could be a limitation in the present study, but all participants felt comfortable in the experimental room with the range of air temperature (22~24 °C). In addition, it is reported that IBL is affected at higher air temperature of 26 °C [1, 4, 5].

In summary, we found that BMI may play a role as an explanatory factor in self-identified cold tolerance and IBL, but no direct relationship was found between self-identified cold tolerance and IBL. This requires further investigating the significant roles of physical characteristics of the human body on body heat exchange and psychological polymorphism in thermal preference.
